# Neonatal Colonic Inflammation Increases Spinal Transmission and Cystathionine β-Synthetase Expression in Spinal Dorsal Horn of Rats with Visceral Hypersensitivity

**DOI:** 10.3389/fphar.2017.00696

**Published:** 2017-10-04

**Authors:** Liting Zhao, Ying Xiao, Rui-Xia Weng, Xuelian Liu, Ping-An Zhang, Chuang-Ying Hu, Shan P. Yu, Guang-Yin Xu

**Affiliations:** ^1^Key Laboratory of Translational Research and Therapy for Neuro-Psycho-Diseases, Laboratory of Translational Pain Medicine, Institute of Neuroscience, The Second Affiliated Hospital of Soochow University, Suzhou, China; ^2^Department of Anesthesiology, Emory University School of Medicine, Atlanta GA, United States

**Keywords:** hydrogen sulfide, visceral pain, spinal cord dorsal horn, excitatory post-synaptic current, irritable bowel syndrome

## Abstract

Irritable bowel syndrome (IBS) is a common gastrointestinal disorder characterized by chronic abdominal pain and alteration of bowel movements. The pathogenesis of visceral hypersensitivity in IBS patients remains largely unknown. Hydrogen sulfide (H_2_S) is reported to play an important role in development of visceral hyperalgesia. However, the role of H_2_S at spinal dorsal horn level remains elusive in visceral hypersensitivity. The aim of this study is designed to investigate how H_2_S takes part in visceral hypersensitivity of adult rats with neonatal colonic inflammation (NCI). Visceral hypersensitivity was induced by neonatal colonic injection of diluted acetic acid. Expression of an endogenous H_2_S synthesizing enzyme cystathionine β-synthetase (CBS) was determined by Western blot. Excitability and synaptic transmission of neurons in the substantia gelatinosa (SG) of spinal cord was recorded by patch clamping. Here, we showed that expression of CBS in the spinal dorsal horn was significantly upregulated in NCI rats. The frequency of glutamatergic synaptic activities in SG was markedly enhanced in NCI rats when compared with control rats. Application of NaHS increased the frequency of both spontaneous and miniature excitatory post-synaptic currents of SG neurons in control rats through a presynaptic mechanism. In contrast, application of AOAA, an inhibitor of CBS, dramatically suppressed the frequency of glutamatergic synaptic activities of SG neurons of NCI rats. Importantly, intrathecal injection of AOAA remarkably attenuated visceral hypersensitivity of NCI rats. These results suggest that H_2_S modulates pain signaling likely through a presynaptic mechanism in SG of spinal dorsal horn, thus providing a potential therapeutic strategy for treatment for chronic visceral pain in patients with IBS.

## Introduction

Irritable bowel syndrome (IBS) is a functional gastrointestinal disorder affecting approximately 20% of populations worldwide (Sandler et al., 1984; Drossman et al., 2002). One of its features is chronic abdominal pain that is difficult to treat for clinicians. There is short of effective therapeutics for the chronic visceral pain and the exact causes and pathogenesis of IBS are not clear. Many animal models are used to mimic the main pathophysiological features of IBS in humans, one of which is neonatal colonic inflammation (NCI) simulating the effect of early life trauma such as acute bacterial gastroenteritis on the development of visceral sensory (Winston et al., 2007; Xu et al., 2008, 2009), which has been reported to generate visceral hyperalgesia in adult rats (Winston et al., 2007; Qu et al., 2013). Therefore, NCI was used in the present study as an animal model to investigate mechanisms of visceral hypersensitivity of IBS.

Hydrogen sulfide (H_2_S), as one of endogenously occurring gasses, was generated from L-cysteine by endogenous enzymes such as cystathionine β-synthetase (CBS), cystathionine-γ-lyase (CSE) and 3-mercaptopyruvate sulfurtransferase (Fukami and Kawabata, 2015; Bian et al., 2016). H_2_S has two types of forms *in vivo*: 1/3 in H_2_S gas form and 2/3 in the form of HS^-^. NaHS is dissociated into Na^+^ and HS^-^, which combined with the H^+^ generating H_2_S, and a dynamic balance between H_2_S and NaHS (Lin et al., 2014). NaHS, as a donor for H_2_S, is used in many studies (Xu et al., 2008; Qu et al., 2013). It was reported that CBS-H_2_S signaling played an important role in modulating visceral sensitivity (Xu et al., 2008) and somatic sensation (Maeda et al., 2009; Yan et al., 2016). However, the roles were controversial. NaHS was reported to alleviate chronic neuropathic pain in rats with chronic constriction injury (Lin et al., 2014) and to inhibit inflammatory hypernociception (Cunha et al., 2008) in mice as well as to attenuate nociception induced by colorectal distention in adult healthy rats and those after colitis (Distrutti et al., 2006a). However, systemic administration of AOAA, an inhibitor of H_2_S synthase, reversed chronic visceral hyperalgesia in adult rats with NCI (Qu et al., 2013) rather than enhancing nociception (Distrutti et al., 2006a). It is proved that the visceral hyperalgesia of rats with NCI is associated with the up-regulated expression of CBS and the increased excitability of dorsal root ganglion (DRG) neurons (Qu et al., 2013). Additionally, it is also proved that the up-regulated expression of CBS and increase in excitation of DRG neurons caused visceral hyperalgesia in adult rats with heterotypic intermittent stress (Wang et al., 2012) as well as hyperalgesia in other animal models, such as neonatal maternal deprivation (Li et al., 2012) and diabetes (Zhang et al., 2013). Therefore, the role of H_2_S in peripheral nervous system seems mainly pro-nociceptive. The analgesia effect of systemic administration of NaHS should be related with the modulation from spinal cord (SC) and/or supraspinal cord. There is little evidence to show the roles of H_2_S in the central nerve system. Many primary afferent neurons with unmyelinated and thinly myelinated fibers projects to the substantia gelatinosa (SG, lamina II) of the dorsal horn of SC (Lu and Perl, 2003; Xie et al., 2017). Since much of the afferent signaling by those fibers is nociceptive signals, it is widely speculated that the SG takes an important role in pain mechanisms (Lu and Perl, 2003; Duan et al., 2017). CBS and CSE are also expressed in the SC (Distrutti et al., 2006a). It is reported that a decrease in spinal H_2_S is pro-nociceptive in the formalin test (Lee et al., 2008); the reduction of H_2_S in the SC during diabetes development leads to a hypersensitivity state in neuropathic rats (Velasco-Xolalpa et al., 2013). However, the role of H_2_S at low concentration in SC is pro-nociceptive in LPS-induced mechanical inflammatory hypernociception (Cunha et al., 2008). So far, it is not clear how the H_2_S in the SC is involved in the regulation of visceral pain.

In the present study, we aimed to clarify the role of H_2_S in SC in a rat model of NCI-induced visceral hypersensitivity. The expression of CBS and CSE in SC was examined by Western blotting techniques from control and NCI rats. Changes in excitatory synaptic transmission were determined using patch-clamp recordings on SG neurons of SC slices. The effect of H_2_S on synaptic transmission and visceral hypersensitivity were explored pharmacologically.

## Materials and Methods

### Animals

Male Sprague Dawley (SD) rats, 150∼200 g body weight, were housed in plastic cages and under controlled conditions (a 12 h light–dark cycle from 8:00 to 20:00, room temperature: 24 ± 2°C) with a standard rodent diet and fresh water. Care and handling of these animals were approved by the Institutional Animal Care and Use Committee of the Soochow University and were strictly in accordance with the guidelines of the International Association for the Study of Pain. All efforts were made to minimize the suffering and the number of animals. Visceral hypersensitivity was induced by neonatal colonic injection of diluted acetic acid, as described previously (Winston et al., 2007; Xu et al., 2008, 2009). In short, 10-day-old pups received an infusion of 0.5% acetic acid solution in 0.2 ml into the colon 2 cm from the anus. Control rats received an equal volume of normal saline (NS). Experiments were performed on these rats at 6∼8 weeks of age.

### Spinal Cord Slice Preparation

Acute SC slices were prepared from adult rats as described previously (Yoshimura and Nishi, 1993; Yang et al., 2001; Liu et al., 2011). The experiments were performed on the colon-related section of SC marked by the afferent nerve fibers of T13-L2 DRGs. The rat was anaesthetized with chloral hydrate (0.4 g/kg, i.p.) and the depth of anesthesia was evaluated by checking the palpebral reflex of animals, and then made an incision on the back. After lumbosacral laminectomy at the level of T13 to L2, the exposed SC was immediately humidified with ice-cold Kreb solution and removed into pre-oxygenated cold Krebs solution (0–4°C) with 95% O_2_ and 5% CO_2_ in a dish. The rats were then killed by exsanguination. The composition of Krebs solution was as follows (in mM): 95 NaCl, 1.8 KCl, 1.2 KH_2_PO_4_, 0.5 CaCl_2,_ 7 MgSO_4,_ 26 NaHCO_3,_ 15 glucose and 50 sucrose, at pH of 7.3–7.4 and an osmolarity of 310–320 mOsm. The ventral, dorsal roots and the pia-arachnoid membrane were removed using microscissors and microforceps under a stereo light microscope. The SC was placed in a shallow groove on an agar block, and the agar block was mounted on the vibrating microslicer stage with cyanoacrylate adhesive. Several transverse slices (450 μm thickness) were cut with a Vibratome (Leica, VT1200S, Germany) while the SC was immersed in cold Krebs solution. The slices were transferred directly to oxygenated Krebs solution at 31°C until use.

### Patch-Clamp Recordings from SG Neurons

After pre-incubation for 1 h (Yoshimura and Nishi, 1993), one slice was placed on a nylon mesh in the recording chamber and fixed with a piece of U-shaped flattened platinum wire with a parallel array of fine nylon threads on top. The slice was continuously perfused with oxygenated recording solution under room temperature at the speed of 10–15 ml/min. The composition of recording solution was as follows (in mM): 127 NaCl, 1.8 KCl, 1.2 KH_2_PO_4_, 2.4 CaCl_2_, 1.3 MgSO_4_, 26 NaHCO_3_ and 15 glucose, at pH of 7.3–7.4 and osmolarity of 300–310 mOsm. The substantia gelatinosa (SG; lamina II) can be indentified as a translucent band across the dorsal horn under the 5x objective (NA 0.10) of an upright microscope (BX51WI, Olympus, Japan). The neurons of SG can be visualized under a 40x magnification water-immersion objective (NA 0.80) with the help of infrared differential interference contrast (IR-DIC) optics. The image of the slice was enhanced with a CCD camera (IR1000E, DAGE MTI) and was displayed on a computer monitor. The patch pipettes were made by a Puller (Sutter-P97, United States). The composition of internal solution of the patch pipette was as follows (in mM): 140 K-Gluconate, 3 KCl, 10 HEPES, 0.2 EGTA, 4 NaCl, and 2 Ma-ATP. Whole-cell voltage-clamp recordings were made from SG neurons as previously described (Yasaka et al., 2007). The bright and well-shaped cells in SG were chosen. The tip (3∼8 MΩ) was driven down to the slice by a micromanipulator (MP-225). After giga ohm (GΩ) seals (usually 2∼8 GΩ) were formed and the whole-cell configuration was obtained, neurons were tested if the resting membrane potential was more negative than -50 mV and direct depolarizing current injections (0∼200 pA, step 40 pA, duration 500 ms) evoked action potentials (APs) overshooting 0 mV when recording excitatory post-synaptic currents (EPSCs) and Aps (Yang and Li, 2000). The resting membrane potential was determined immediately after cell membrane rupture (Cui et al., 2011). The neurons were holding at -70 mV for recording spontaneous excitatory post-synaptic currents (sEPSCs). Miniature EPSCs (mEPSCs) were recorded in the presence of TTX (1 μM) in the recording solution. Although H_2_S could be produced endogenously, it is presumed to exist at very low concentrations in animal tissues due to its toxicity (Qu et al., 2008). Therefore, NaHS was used at 2.5 μM in the present study. CNQX (10 μM) was also applied in some experiments to identify the property of EPSC. Drugs were dissolved in ACSF from stocks on the day of experiment and added by perfusion. Signals were acquired using a Multiclamp 700B amplifier, Digidata 1440A interface and ClampEx10.3 software (Molecular Devices, Axon, United States) and filtered at 5 kHz with Bessel filter of amplifier. Data were stored on a computer for offline analysis. The data of inhibitory neurons were not included in the present analysis. The inhibitory neurons can be classified as its tonic firing pattern (Cui et al., 2011). In all cases, n refers to the number of neurons recorded.

### Real-time qPCR

Total RNA was exacted from the dorsal horn of T13-L2 SC in control and NCI rats by Trizol method. cDNA was synthesized from total RNA using EasyScript First-Strand cDNA Synthesis SuperMix kit (Transgen Biotech) following the instructions. qPCR was conducted according the protocol of TransStart Tip Green qPCR SuperMix kit (Transgen Biotech). Negative control reactions were performed by omitting cDNA temple. The relative expression level for each target gene was normalized via 2^-ΔΔCt^ method.

### Western Blotting

The expression of CBS in spinal dorsal horn corresponding to afferent nerve fibers from DRGs (T_13_-L_2_) for adult NCI and control rats (6–8 week old) were determined using western blot analyses as described previously (Qu et al., 2008). In short, the tissue of spinal dorsal horn were lyzed in radioimmunoprecipitation assay buffer containing 1% NP-40, 0.5% Na deoxycholate, 0.1% SDS, PMSF (10 μl/ml) and aprotinin (30 μl/ml; Sigma). The lysates were then microfuged for 30 min at 4°C. After fractionating of SC protein extract on 4 and 10% polyacrylamide gels, proteins were transferred to polyvinylidene difluoride (PVDF) membranes (Millipore). Membranes were then blocked in Tris-buffered saline (TBS) containing 5% dilution of non-fat milk powder under room temperature. Membrane of 55–72 KD was incubated with anti-CBS antibody (1:1000; Abnova, Taiwan, China) and the membrane of 35–55 KD was incubated with anti-GAPDH antibody (1:1000; Goodhere, China) under 4°C overnight in TBS containing 1% milk. After washed in TBS containing 0.5% Tween-20 (TBST), membranes were incubated with horseradish peroxidase (HRP)-conjugated secondary antibodies (1:4000; Chemicon) in TBS containing 1% milk at room temperature. The membrane was then washed with TBST and the immunoreactive proteins were detected by enhanced chemiluminescence (ECL kit; AmershamBiosciences, Arlington Heights, IL, United States) and appropriate exposure to chemiluminescent imaging system (ChemiDoc XRS, Biorad). Band intensities were measured using Image J software. All samples were normalized to GAPDH as loading control.

### Drug Application

For behavioral experiments, *O*-(carboxymethyl)hydroxylamine hemihydrochloride (AOAA, an inhibitor of CBS) was purchased from Sigma-Aldrich and freshly prepared in NS (0.9% wt/vol NaCl). At the age of 5 week, AOAA was injected intrathecally at 10 μg/kg body weight, one time per day for a consecutive 7 days as described previously (Qu et al., 2013).

### Statistical Analysis

A fixed length of trace (5 min) that contains more than 300 events was analyzed using MiniAnalysis program 6.0.3 (Synaptosoft) for frequency and amplitude distributions of miniature and spontaneous EPSCs. After the automatical detection for peaks, each detected event was visually checked to exclude the false data. Three to four times the root mean square value of its background noise was set to be the detection threshold for an event in a set of data. Cumulative fractions were calculated before and after an addition of a drug. Kolmogorov–Smirnov test was used to analyze EPSCs. Data are presented as the means ± SEM. Error bars in the figures stand for SEM. Normality was checked for all data before using the Mann–Whitney test as *post hoc* test following Friedman ANOVA, two-sample *t*-test and paired sample Wilcoxon signed rank test with Origin 8 (Origin Lab, Inc., United States), as appropriate. Statistical significance was determined as *P* < 0.05.

## Results

### Upregulated Expression of CBS in Spinal Cord of NCI Rats

**FIGURE 1:**
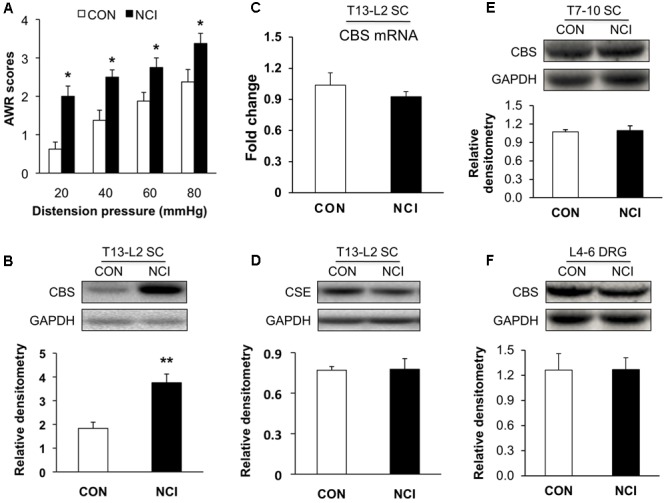
Upregulated expression of CBS in spinal cord (SC) of NCI rats. **(A)** NCI treatment significantly increased AWR scores to CRD at age of 6 weeks compared with the age-matched control rats (*n* = 8). **(B)** Increase in expression of CBS in SC at T13-L2 levels from NCI rats at age of 6 week compared with control (CON) rats (*n* = 5 for control group and *n* = 8 for NCI group). **(C)** No difference of CBS mRNA expression in SC between CON and NCI rats at age of 6 weeks (*n* = 5). **(D)** No difference of CSE expression in SC at T13-L2 level between CON and NCI rats at age of 6 weeks (*n* = 5). **(E)** No difference of CBS expression in SC at T7-T10 level between CON and NCI rats at age of 6 weeks (*n* = 4). **(F)** No difference of CBS expression in dorsal root ganglion (DRG) at L4-L6 level between CON and NCI rats at age of 6 weeks (*n* = 4). ^∗^*P* < 0.05, ^∗∗^*P* < 0.01 when compared with CON.

CVH was determined by measuring AWR scores in response to colorectal distention (CRD) at age of 6 week for control (CON, *n* = 8) and NCI (*n* = 8) rats. AWR scores were significantly higher for NCI rats at different distention pressures (20, 40, 60, and 80 mmHg) when compared with those of age-matched controls (**Figure 1A**, Control group: 0.63 ± 0.18, 1.38 ± 0.26, 1.88 ± 0.23, 2.38 ± 0.32 for 20, 40, 60, and 80 mmHg, respectively; NCI group: 2.00 ± 0.27, 2.5 ± 0.19, 2.75 ± 0.25, 3.38 ± 0.26 for 20, 40, 60, and 80 mmHg, respectively; ^∗^*P* < 0.05, for the same pressure, use Mann–Whitney test as *post hoc* test following Friedman ANOVA). It suggested that NCI significantly induced visceral hypersensitivity in adult rats. This result was consistent with the previous report from our group (Qu et al., 2013).

Since H_2_S is an important mediator in both peripheral and central nervous systems in various types of pain, and its synthase CBS, which has much higher activity than CSE (Distrutti et al., 2006a), is expressed in SC. Western blotting assays were performed to determine whether expression of CBS was changed in SC after NCI. Anti-CBS antibody labeled a ∼60 kDa molecular mass protein (**Figure 1B**). At 6 weeks of the age, relative densitometry of CBS was 1.83 ± 0.26 (*n* = 5) for control and 3.76 ± 0.37 (*n* = 8) for NCI group, respectively. It suggested that the expression of CBS was significantly upregulated after NCI (**Figure 1B**, ^∗∗^*P* < 0.01, two-sample *t*-test). However, the mRNA level of CBS was not significantly altered after NCI (**Figure 1C**, *n* = 5 for each group). Also, there was no change in the expression of CSE (∼70 kDa) in SC between control and NCI group (**Figure 1D**, *n* = 5 for each group, *P* > 0.05, two-sample *t*-test; 0.77 ± 0.03 for control; 0.78 ± 0.08 for NCI). The expression of CBS in T7-10 SC (*n* = 4, **Figure 1E**) and at L_4-6_ DRGs (*n* = 4, **Figure 1F**) was not altered after NCI. Thus, NCI remarkably increased CBS expression in SC corresponding to the afferents of T_13_-L_2_ DRGs when compared with that of age-matched control rats.

### NCI Enhanced the Excitation of SG Neurons

**FIGURE 2:**
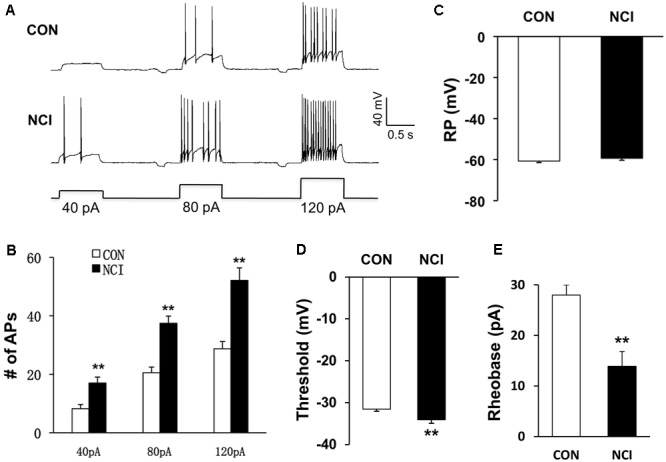
NCI increased the excitation of SG neurons. **(A)** Representative traces of APs caused by three different depolarizing current stimulation (40, 80, and 120 pA) in SG neurons from CON and NCI rats. **(B)** NCI greatly increased the frequency of APs elicited by current injection of 40, 80, and 120 pA compared with controls. *n* = 17 for control group and *n* = 18 for NCI group, ^∗^*P* < 0.05 vs. CON, ^∗∗^*P* < 0.01 vs. CON. **(C)** The mean resting membrane potential (RP) had no significant difference between CON and NCI rats (*n* = 59 for control group and *n* = 33 for NCI group). **(D)** There was significant hyperpolarization in action potential (AP) threshold between CON and NCI rats. *n* = 35 for control group and *n* = 20 for NCI group, ^∗∗^*P* < 0.01. **(E)** NCI significantly decreased the rheobase when compared with CON. *n* = 35 for control group and *n* = 20 for NCI group, ^∗∗^*P* < 0.01.

Whether the intrinsic excitatory property of SG neurons was changed by NCI was examined by whole-cell patch-clamp recordings in acutely prepared transverse lumbar SC slices from control and NCI rats. Among the SG neurons recorded (*n* = 109, 70 from control slices, 39 from NCI slices), different discharge patterns were observed, including tonic-firing, delayed-firing, gap-firing, initial-bursting, bursting and phasic firing, as previously reported (Yoshimura and Jessell, 1989; Ruscheweyh and Sandkuhler, 2002). SG neurons recorded from control (15.7%, 11/70) and from NCI group (15.4%, 6/39) belong to tonic-firing neurons. Since most inhibitory neurons are reported to show tonic-firing pattern (Cui et al., 2011), the SG neurons who showed tonic-firing pattern was excluded from later analysis, and the data from neurons of other firing patterns were pooled together to analyze the property of excitatory neurons. The representative traces for three different current stimuli from two typical neurons illustrated that the excitation of SG neurons was increased by NCI (**Figure 2A**). The average results were shown in **Figure 2B**. The number of APs of control SG neurons (*n* = 17) under 40, 80, and 120 pA current stimulation was 8.23 ± 1.45, 20.53 ± 1.93, and 28.73 ± 2.47, respectively. The number of APs of NCI SG neurons (*n* = 18) under 40, 80, and 120 pA current stimulation was 17.00 ± 2.04, 37.44 ± 2.48, and 52.12 ± 4.29, respectively. It suggested that the excitability of SG neurons was significantly increased by NCI (**Figure 2B**; ^∗∗^*P* < 0.01; two sample *t*-test for each current stimulation). In addition, the mean resting membrane potential for CON neurons was -60.71 ± 0.73 mV (*n* = 59) and -59.36 ± 0.95 mV (*n* = 33) for NCI neurons. They did not differ significantly (**Figure 2C**, *P* > 0.05, two sample *t*-test). However, the AP threshold for NCI neurons (-34.11 ± 0.84 mV, *n* = 35) significantly hyperpolarized when compared with that of the CON neurons (-31.56 ± 0.53 mV, *n* = 20, **Figure 2D**, ^∗∗^*P* < 0.01, two sample *t*-test). Rheobase reflects the minimal injected current to evoke an AP. The rheobase of SG neurons was 13.89 ± 2.88 pA and 28.00 ± 1.99 pA for NCI and CON group, respectively. It suggested that NCI dramatically decreased the rheobase (**Figure 2E**, ^∗∗^*P* < 0.01, two sample *t*-test), which is consistent with the increased excitation of SG neurons by NCI.

### NCI Enhanced Spontaneous Excitatory Neurotransmission of SG Neurons

**FIGURE 3:**
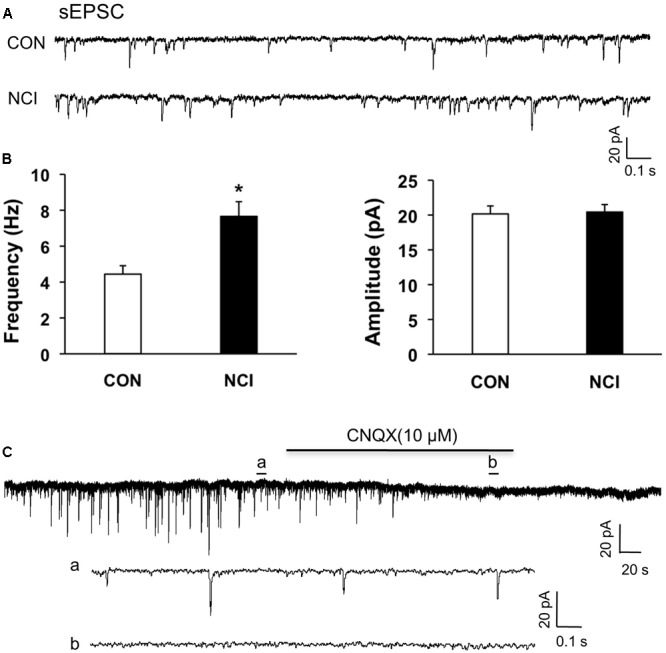
NCI increased the frequency of sEPSCs in SG neurons. **(A)** Representative traces of spontaneous excitatory post-synaptic currents (sEPSC) recorded from SG neurons hold at –70 mV in voltage clamp from control (CON) and NCI rats. **(B)** NCI increased the frequency of sEPSC (left, ^∗^*P* < 0.05, compared with CON) but failed to change the amplitude of sEPSC (right) when compared with controls (*n* = 29 for control group and *n* = 35 for NCI group). **(C)** The administration of CNQX (10 μM) blocked all the sEPSCs in SG neurons from control rats. a: An expanded trace for sEPSC recorded in the absence of CNQX. b: An expanded trace for sEPSC recorded in the presence of CNQX.

Since the excitation of SG neurons was enhanced by NCI, the excitatory neurotransmission was presumed to be enhanced by NCI as well. The sEPSCs of SG neurons were then compared between control and NCI group. The representative traces from two typical neurons of control and NCI slices illustrated an increase in frequency of sEPSCs of SG neuron in NCI group (**Figure 3A**). The average results were also shown in **Figure 3B**. Mean frequency of sEPSCs averaged to 4.44 ± 0.74 Hz in control group and 7.66 ± 1.17 Hz in NMD group, respectively (**Figure 3B** left, *n* = 29 for control, *n* = 35 for NCI, ^∗^*P* < 0.05, two sample *t*-test). In parallel, the mean peak amplitude of sEPSCs averaged to 20.16 ± 1.13 pA in control group and 20.44 ± 1.02 pA in NMD group, respectively (**Figure 3B**, right, *n* = 29 for control, *n* = 35 for NCI, *P* > 0.05, two sample *t*-test). The sEPSCs can be completely blocked by CNQX (10 μM, **Figure 3C**). It suggested that the AMPA receptor-mediated glutamatergic synaptic activity was significantly enhanced in SG of NCI rats.

### NaHS Enhanced sEPSCs of SG Neurons in Control Slices

**FIGURE 4:**
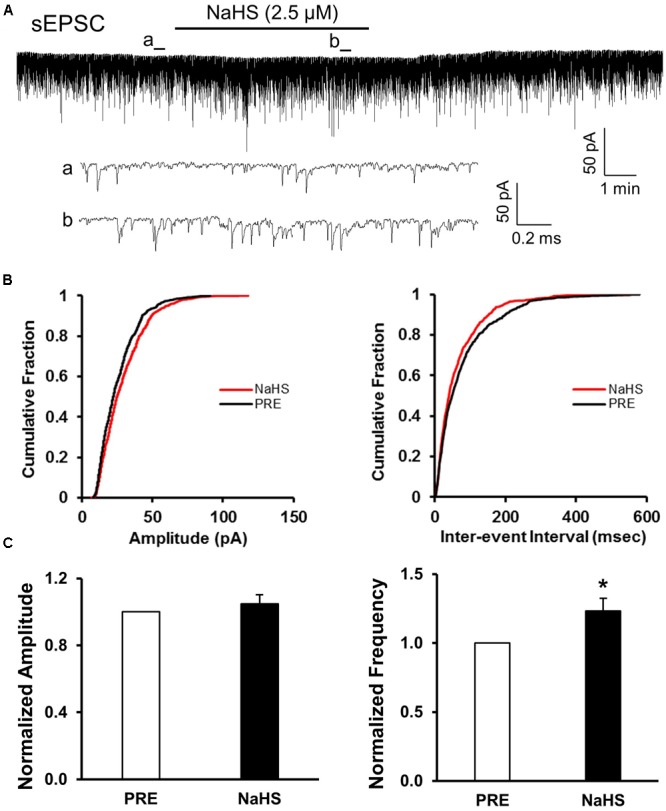
NaHS enhanced the frequency of sEPSCs in control group. **(A)** Representative current traces of sEPSCs recorded in the presence and absence of NaHS (2.5 μM). a: An expanded trace for sEPSC recorded in the absence of NaHS; b: a expanded trace for sEPSC recorded in the presence of NaHS. **(B)** Cumulative distribution analysis of amplitude and inter-event intervals from one representative SG neuron. NaHS had no discernible effect on the amplitude, but NaHS caused a notably shift toward shorter inter-event intervals (*n* = 10, *P* < 0.05, Kolmogorov–Smirnov test). **(C)** Bar plot showing NaHS significantly increased mean frequency of sEPSC without change in peak amplitude. *n* = 9, ^∗^*P* < 0.05.

Since CBS were upregulated and the excitability of SG was increased in NCI group, we presumed that NaHS, a donor of H_2_S, could increase the excitatory neurotransmission of SG in SC slices of control rats. NaHS (2.5 μM) were perfused for 5 min after recording the pretreated data for at least 5 min. The two enlarged traces of a typical current trace illustrated the increase in frequency of sEPSCs in a neuron of SG in control slices (**Figure 4Aa,b**). NaHS had no significant effect on the amplitude of sEPSCs (**Figures 4B,C**, left; *P* > 0.05, paired sample Wilcoxon signed rank test). Mean amplitudes averaged to 23.21 ± 1.58 pA before and 24.1 ± 1.73 pA after addition of NaHS (104.78 ± 5.57%, *n* = 10, *P* > 0.05, paired sample Wilcoxon signed rank test). Kolmogorov–Smirnov test proved that NaHS shifted the cumulative fraction of inter-event intervals of sEPSCs toward smaller value (**Figure 4B**, right) and increased the mean frequency of sEPSC significantly (**Figure 4C**, right; *n* = 9, ^∗^*P* < 0.05, paired sample Wilcoxon signed rank test). There was a significant increase in average frequency from 5.53 ± 1.63 Hz before, to 6.31 ± 1.88 Hz after the addition of NaHS. The average increase achieved 123.24 ± 9.13%. It suggested that NaHS application increased the spontaneous glutamatergic synaptic activity of SG neurons in control rats.

### Presynaptic Effect of NaHS

**FIGURE 5:**
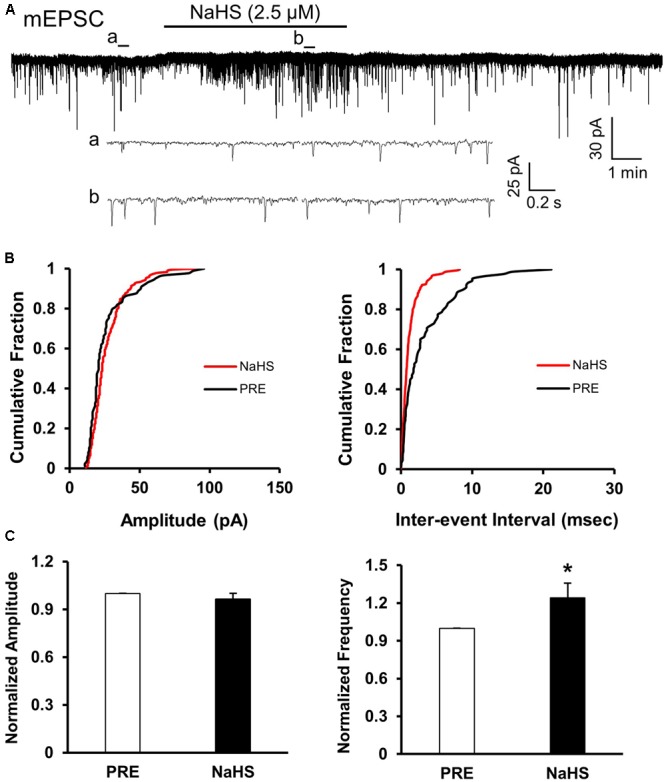
NaHS enhanced the frequency of mEPSCs in control group. **(A)** Representative current traces of mEPSCs recorded in the presence and absence of NaHS (2.5 μM). Na^+^ channel blocker TTX (1 μM) was used in Kreb solution. a: An expanded trace for mEPSC recorded in the absence of NaHS; b: an expanded trace for mEPSC recorded in the presence of NaHS. **(B)** Cumulative distribution analysis of peak amplitude and inter-event intervals from the same neuron showed that NaHS reduced the inter-event intervals of mEPSC (*n* = 10, *P* < 0.01, Kolmogorov–Smirnov test) but had no effect on the amplitude. **(C)** Bar plot showing NaHS significantly increased mean frequency of sEPSCs but not amplitude. *n* = 10, ^∗^*P* < 0.05.

To assess whether presynaptic or post-synaptic mechanism underlies the effect of NaHS in SG, we used a well-established method, the quanta analysis of miniature EPSCs. The frequency and amplitude of mEPSCs of SG from control rats were measured before and after application of NaHS (2.5 μM). The typical trace and its two expanded traces of a representative neuron showed that NaHS increased the frequency of mEPSCs recorded in SG neurons of control slices (**Figure 5A**). NaHS didn’t significantly affect the amplitude of mEPSCs (**Figures 5B,C**, left). Mean amplitudes averaged to 25.96 ± 1.87 pA before and 24.53 ± 0.99 pA after addition of NaHS (96.49 ± 3.5%, *n* = 10, *P* > 0.05, paired sample Wilcoxon signed rank test). Kolmogorov–Smirnov test proved that NaHS shifted the cumulative fraction of inter-event intervals of mEPSCs toward smaller value (**Figure 5B**, right) and significantly increased the mean frequency of mEPSC (**Figure 5C**, right, *n* = 10, ^∗^*P* < 0.05, paired sample Wilcoxon signed rank test). There was a significant increase in average frequency from 4.51 ± 0.5 Hz before, to 5.56 ± 0.67 Hz after the addition of NaHS. The average increase achieved 124.14 ± 11.58%. It is well-known that change in frequency of mEPSCs reflects the increase of presynaptic neurotransmitter release, whereas change in amplitude of mEPSCs reflects the changes at the post-synaptic membrane (Malgaroli and Tsien, 1992; Yoshimura and Nishi, 1993). The result of mEPSCs suggested that the effect of NaHS application in control slices is most likely presynaptic.

### AOAA Treatment Reversed the Enhanced sEPSCs and Visceral Hypersensitivity

**FIGURE 6:**
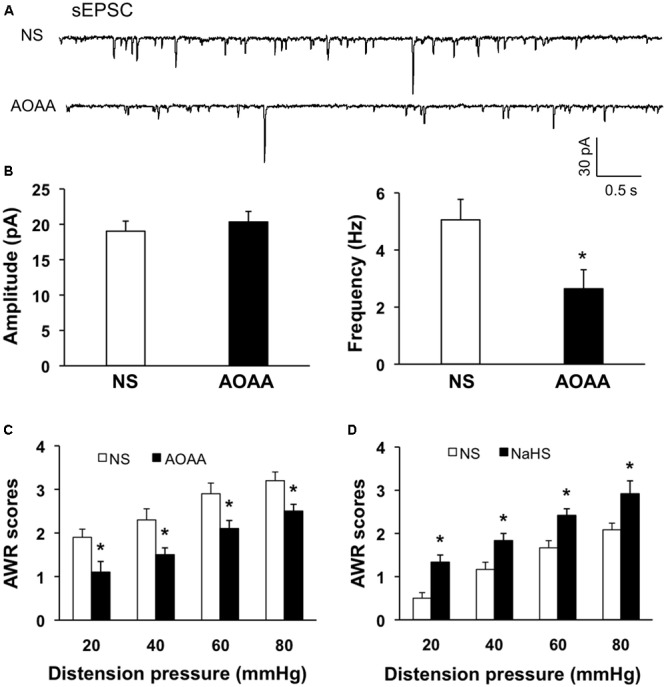
AOAA decreased the frequency of sEPSCs in SG neurons and attenuated visceral pain of NCI-rats. **(A)** Representative traces of sEPSCs recorded from SG neurons holding at –70 mV in voltage clamp from NCI and AOAA-treated rats. **(B)** AOAA treatment didn’t change the amplitude of sEPSC compared with NCI rats. The frequency of sEPSC was decreased after AOAA treatment compared with NCI rats. *n* = 10 for NS, *n* = 9 for AOAA, ^∗^*P* < 0.05. **(C)** Bar plot showing that intrathecal injection of AOAA significantly decreased AWR scores of NCI rats. *n* = 5, ^∗^*P* < 0.05. **(D)** Bar plot showing that intrathecal injection of NaHS significantly increased AWR scores of control rats. *n* = 6, ^∗^*P* < 0.05.

Since CBS were upregulated and the excitability of SG was increased in NCI rats, we hypothesized that the increase of excitability of SG in NCI rats might be associated with the upregulation of CBS. Therefore, inhibition of CBS was hypothesized to decrease excitability of SG in NCI rats. AOAA, an inhibitor of CBS, was intrathecally injected in the present study to determine the effect of inhibiting CBS on sEPSCs of SG neurons in NCI slices. Data were compared with the sEPSCs of SG neurons in slices from NS-treated NCI rats. The representative traces from two typical neurons of NS and AOAA treated group illustrated a decrease in frequency of sEPSCs of SG neuron in AOAA group (**Figure 6A**). The average results were also shown in **Figure 6B**. The mean peak amplitude of sEPSCs averaged to 19.03 ± 1.42 pA in NCI group and 20.33 ± 1.48 pA in AOAA group, respectively (**Figure 6B** left, *n* = 10 for NS, *n* = 9 for AOAA, *P* > 0.05, two sample *t*-test). In parallel, mean frequency of sEPSCs averaged to 5.05 ± 0.71 Hz in NCI group and 2.64 ± 0.66 Hz in AOAA group, respectively (**Figure 6B** right, *n* = 10 for NS, *n* = 9 for AOAA, ^∗^*P* < 0.05, two sample *t*-test). It suggested that the glutamatergic synaptic activity was significantly decreased in SG of AOAA-treated NCI rats.

We then detected the effect of AOAA treatment on visceromotor responses to CRD in NCI rats. Intrathecal injection of AOAA significantly attenuated AWR scores at different distention pressures in NCI rats when compared with NS group (**Figure 6C**, *n* = 5 for each group, ^∗^*P* < 0.05, use Mann–Whitney test as *post hoc* test following Friedman ANOVA; NS group: 1.9 ± 0.19, 2.3 ± 0.25, 2.9 ± 0.24, 3.2 ± 0.2 for 20, 40, 60, and 80 mmHg, respectively; AOAA group: 1.1 ± 0.24, 1.5 ± 0.16, 2.1 ± 0.19, 2.5 ± 0.16 for 20, 40, 60, and 80 mmHg, respectively). These data proved that AOAA treatment reversed the visceral hypersensitivity in NCI rats. It is in compliance with the result of our previous study (Qu et al., 2013).

Since NaHS/H_2_S was proved to increase the glutamatergic synaptic transmission of SG neurons of control slices in the present study. We presumed that NaHS application could induce visceral hypersensitivity of control rats as well. NaHS solution (2.5 μM) was intrathecally injected into SC (L_5-6_) for consecutive 7 days and the behavioral responses were assessed. The AWR scores for all the distension pressure are significantly higher than the corresponding values of NS-treated rats (**Figure 6D**, *n* = 6, ^∗^*P* < 0.05, Mann–Whitney test as *post hoc* test following Friedman ANOVA; NS, 0.5 ± 0.16, 1.1 ± 0.19, 1.7 ± 0.2, 2.1 ± 0.19 for 20, 40, 60, and 80 mmHg, respectively; NaHS, 1.4 ± 0.19, 1.9 ± 0.19, 2.5 ± 0.16, 3.1 ± 0.29 for 20, 40, 60, and 80 mmHg, respectively). These data demonstrate that NaHS/H_2_S induced hyperalgesic effect in healthy rats, which supports the pronociceptive effect of CBS upregulation in NCI rats.

## Discussion

The pathogenesis of chronic visceral pain in patients with IBS remains largely unknown. The present study demonstrates that hydrogen sulfide signaling at SC level produces visceral hypersensitivity in adult rats with NCI. The conclusion is based on the following observations: (1) NCI leads to a significant increase in expression of CBS in SC where receives input from T13-L2 DRG neurons (**Figure 1B**); (2) NCI significantly increases neuronal excitability and excitatory synaptic transmission in SG of spinal dorsal horn (**Figures 2, 3**); (3) Intrathecal injection of AOAA (the antagonist of CBS) not only reversed the visceral hyperalgesia of rats with NCI but also decreased excitatory synaptic transmission in SG (**Figure 6**); (4) Intrathecal injection of NaHS (2.5 μM) not only led to visceral pain but also significantly increases excitatory synaptic transmission of SG in control rats (**Figures 4, 5**). These results indicate that upregulation of CBS expression contributed to enhanced synaptic transmission at SG neurons and visceral hypersensitivity of adult rats with NCI.

Although much attention has been drawn to study the roles of H_2_S, there is yet no consensus on this topic. Reports on H_2_S in pain processing are controversial. It is reported that H_2_S at physiological levels in SC plays an anti-nociceptive role through inhibition of microglial function both in formalin-induced mechanical pain (Lee et al., 2008) and in neuropathic pain of rats during diabetes development (Velasco-Xolalpa et al., 2013). Hydrogen sulfide prevents opioid withdrawal-induced pain sensitization (Yang et al., 2014). However, the H_2_S of low concentration in SC in LPS-induced mechanical inflammatory hypersensitivity is pro-nociceptive (Cunha et al., 2008). The role of H_2_S in SG in NCI-induced visceral hyperalgesia is consistent with that in LPS-induced mechanical inflammatory hypernociception, indicating that the modulatory role of H_2_S in SC on pain is attributed to the pain model. In the present study, the increase in frequency of mEPSCs by NaHS, without significant change in amplitude of mEPSCs, strongly suggested that these effects were attributable to the action at presynaptic site of SG (**Figures 4, 5**). The action site of H_2_S is consistent with that in other tissues, such as neuromuscular junction (Gerasimova et al., 2015) and superior mesenteric ganglion (Sha et al., 2013). Since H_2_S increased presynaptic glutamate neurotransmission, upregulation of CBS contributed to the over excitation of SG in NCI rats. It is reported that the effect of H_2_S in mouse neuromuscular junction is mediated by intracellular Ca^2+^ and cAMP signaling and involves presynaptic ryanodine receptors (Gerasimova et al., 2015). The mechanism of H_2_S action on synaptic transmission in SG may be similar to that reported in mouse neuromuscular junction and further studies are definitely needed. Since glial activation is a common mechanism underlying spinal synaptic plasticity (Zhou and Liu, 2017), future investigation into the roles of H_2_S on glial-neuronal interaction is very important.

In the present study, since NaHS or AOAA were injected intrathecally, these drugs may act on SC and/or DRGs. It is therefore difficult to rule out the contribution of DRGs. However, by considering the data obtained by the electrophysiological recordings from the SG neurons of spinal dorsal horn, it is reasonable to hypothesize that SC might be one of the important action sites for H_2_S. CBS and CSE are two important endogenous synthetases for H_2_S. Both CBS and CSE are expressed in the SC (Distrutti et al., 2006a). CBS is reported to be localized to astrocytes-enriched tissues such as hippocampus, temporal lobe, and cortex (Distrutti et al., 2006b; Miyamoto et al., 2015). In SC, CBS might also be localized to astrocytes although it lacks direct evidence. The activity of CBS is reported to be 30-fold greater than that of CSE (Distrutti et al., 2006a). We showed here that CBS in SC is dramatically upregulated while CSE expression was not significantly altered (**Figure 1**), indicating CBS might be a major factor contributing to visceral hyperalgesia of NCI rats. However, the mRNA level of CBS was not altered. Reasons for this discrepancy might include: regulation of CBS expression at mRNA and protein levels may not happen at the same time and the CBS protein degradation speed was changed. In the present study, the upregulation of CBS expression at translational level and/or downregulation of protein degradation speed might be the underlying mechanisms. In addition, the protein level of CBS is not equal to endogenous H_2_S since endogenous H_2_S in the nervous system is generated from CBS, CSE and 3MST.

H_2_S has multiple molecular targets in peripheral tissues and in the CNS. Spinal and peripheral H_2_S activates L-type channel (Tan et al., 2010), T-type Ca^2+^ channels (such as Cav3.2 channel) in the primary afferents and/or spinal nociceptive neurons (Maeda et al., 2009). It also elevates the intracellular Ca^2+^ levels through activation of TRPA1 channels in capsaicin-sensitive DRG neurons (Fukami and Kawabata, 2015), thus leading to L-glutamate release enhancement in SG neurons and sensitization of nociceptive processing (Inoue et al., 2012). Phosphorylation of spinal ERK underlies the development of visceral pain (Matsunami et al., 2012). H_2_S also promotes the activation of NMDA receptors via stimulation of cyclic adenosine monophosphate (cAMP)/protein kinase A signaling and/or inhibition of phosphodiesterase (Fukami and Kawabata, 2015), leading to the facilitation of nociceptive synaptic efficacy in spinal dorsal horn (Wu et al., 2010). On the other hand, some ion channels, receptors and transcription factors are involved in the anti-nociciptive effect of H_2_S. The activation of ATP dependent K^+^ channels (Kawabata et al., 2007) and GABAb receptors (Qu et al., 2008) by H_2_S could result in an increase of K^+^ conductance, which might inhibit visceral nociception in response to colorectal distension in rats (Kawabata et al., 2007). The H_2_S donor Na_2_S was reported to activate the mu-opioid receptor MOR (Syhr et al., 2015). The inhibition of NF-κB by H_2_S could inhibit microglial activation, thus preventing the development of neuropathic pain (Kida et al., 2015). ATF3 and expression of pCREB can be inhibited by H_2_S in the SC to alleviate chronic neuropathic pain (Lin et al., 2014; Kida et al., 2015). The precise mechanism that underlies the present results still needs to be further studied in the future.

## Conclusion

The present study shows that the upregulation of CBS in SC takes part in visceral hypersensitivity of adult rats with NCI through a presynaptic mechanism by increasing excitatory neurotransmission and sensitizing the SG neurons. Inhibiting H_2_S signaling in SC can alleviate the visceral pain induced by CRD. The present results shed light on the role of H_2_S in SC in development of visceral hypersensitivity and provide new insights into the treatment of chronic visceral pain in patients with IBS.

## Ethics Statement

This study was carried out in accordance with the recommendations of the International Association for the Study of Pain (IASP). The protocol was approved by the Institutional Animal Care and Use Committee of Soochow University, China.

## Author Contributions

LZ, YX, and R-XW performed experiments, analyzed data and prepared figures and the manuscript. XL and P-AZ performed experiments and analyzed data. C-YH and SY revised the manuscript. G-YX designed experiments, supervised the experiments and finalized the manuscript. All the authors have read and approved the paper.

## Conflict of Interest Statement

The authors declare that the research was conducted in the absence of any commercial or financial relationships that could be construed as a potential conflict of interest.

## References

[B1] Bian J. S. Olson K. R. Zhu Y. C. (2016). Hydrogen sulfide: biogenesis, physiology, and pathology. *Oxid. Med. Cell. Longev.* 2016:6549625 10.1155/2016/6549625 PMC4842377 27148431

[B2] Cui L. Kim Y. R. Kim H. Y. Lee S. C. Shin H. S. Szabo G. (2011). Modulation of synaptic transmission from primary afferents to spinal substantia gelatinosa neurons by group III mGluRs in GAD65-EGFP transgenic mice. *J. Neurophysiol.* 105 1102–1111. 10.1152/jn.00108.2010 21177998

[B3] Cunha T. M. Dal-Secco D. Verri W. A. Jr. Guerrero A. T. Souza G. R. Vieira S. M. (2008). Dual role of hydrogen sulfide in mechanical inflammatory hypernociception. *Eur. J. Pharmacol.* 590 127–135. 10.1016/j.ejphar.2008.05.048 18585702

[B4] Distrutti E. Sediari L. Mencarelli A. Renga B. Orlandi S. Antonelli E. (2006a). Evidence that hydrogen sulfide exerts antinociceptive effects in the gastrointestinal tract by activating K-ATP channels. *J. Pharmacol. Exp. Ther.* 316 325–335. 10.1124/jpet.105.091595 16192316

[B5] Distrutti E. Sediari L. Mencarelli A. Renga B. Orlandi S. Russo G. (2006b). 5-amino-2-hydroxybenzoic acid 4-(5-thioxo-5H-[1,2]dithiol-3yl)-phenyl ester (ATB-429), a hydrogen sulfide-releasing derivative of mesalamine, exerts antinociceptive effects in a model of postinflammatory hypersensitivity. *J. Pharmacol. Exp. Ther.* 319 447–458. 10.1124/jpet.106.106435 16855178

[B6] Drossman D. A. Camilleri M. Mayer E. A. Whitehead W. E. (2002). AGA technical review on irritable bowel syndrome. *Gastroenterology* 123 2108–2131. 10.1053/gast.2002.37095 12454866

[B7] Duan B. Cheng L. Ma Q. (2017). Spinal circuits transmitting mechanical pain and itch. *Neurosci. Bull.* 10.1007/s12264-017-0136-z [Epub ahead of print]. PMC5799122 28484964

[B8] Fukami K. Kawabata A. (2015). Hydrogen sulfide and neuronal differentiation: focus on Ca^2+^ channels. *Nitric Oxide* 46 50–54. 10.1016/j.niox.2015.02.001 25660006

[B9] Gerasimova E. Lebedeva J. Yakovlev A. Zefirov A. Giniatullin R. Sitdikova G. (2015). Mechanisms of hydrogen sulfide (H2s) action on synaptic transmission at the mouse neuromuscular junction. *Neuroscience* 303 577–585. 10.1016/j.neuroscience.2015.07.036 26192092

[B10] Inoue M. Fujita T. Goto M. Kumamoto E. (2012). Presynaptic enhancement by eugenol of spontaneous excitatory transmission in rat spinal substantia gelatinosa neurons is mediated by transient receptor potential A1 channels. *Neuroscience* 210 403–415. 10.1016/j.neuroscience.2012.02.040 22426238

[B11] Kawabata A. Ishiki T. Nagasawa K. Yoshida S. Maeda Y. Takahashi T. (2007). Hydrogen sulfide as a novel nociceptive messenger. *Pain* 132 74–81. 10.1016/j.pain.2007.01.026 17346888

[B12] Kida K. Marutani E. Nguyen R. K. Ichinose F. (2015). Inhaled hydrogen sulfide prevents neuropathic pain after peripheral nerve injury in mice. *Nitric Oxide* 46 87–92. 10.1016/j.niox.2014.11.014 25461302 PMC4361306

[B13] Lee A. T. Shah J. J. Li L. Cheng Y. Moore P. K. Khanna S. (2008). A nociceptive-intensity-dependent role for hydrogen sulphide in the formalin model of persistent inflammatory pain. *Neuroscience* 152 89–96. 10.1016/j.neuroscience.2007.11.052 18248901

[B14] Li L. Xie R. Hu S. Wang Y. Yu T. Xiao Y. (2012). Upregulation of cystathionine beta-synthetase expression by nuclear factor-kappa B activation contributes to visceral hypersensitivity in adult rats with neonatal maternal deprivation. *Mol. Pain* 8:89 10.1186/1744-8069-8-89 PMC3545973 23249427

[B15] Lin J. Q. Luo H. Q. Lin C. Z. Chen J. Z. Lin X. Z. (2014). Sodium hydrosulfide relieves neuropathic pain in chronic constriction injured rats. *Evid. Based Complement. Alternat. Med.* 2014:514898 10.1155/2014/514898 PMC4260443 25506383

[B16] Liu T. Fujita T. Kumamoto E. (2011). Acetylcholine and norepinephrine mediate GABAergic but not glycinergic transmission enhancement by melittin in adult rat substantia gelatinosa neurons. *J. Neurophysiol.* 106 233–246. 10.1152/jn.00838.2010 21525362

[B17] Lu Y. Perl E. R. (2003). A specific inhibitory pathway between substantia gelatinosa neurons receiving direct C-fiber input. *J. Neurosci.* 23 8752–8758. 14507975 10.1523/JNEUROSCI.23-25-08752.2003 PMC6740424

[B18] Maeda Y. Aoki Y. Sekiguchi F. Matsunami M. Takahashi T. Nishikawa H. (2009). Hyperalgesia induced by spinal and peripheral hydrogen sulfide: evidence for involvement of Cav3.2 T-type calcium channels. *Pain* 142 127–132. 10.1016/j.pain.2008.12.021 19167819

[B19] Malgaroli A. Tsien R. W. (1992). Glutamate-induced long-term potentiation of the frequency of miniature synaptic currents in cultured hippocampal neurons. *Nature* 357 134–139. 10.1038/357134a0 1349728

[B20] Matsunami M. Kirishi S. Okui T. Kawabata A. (2012). Hydrogen sulfide-induced colonic mucosal cytoprotection involves T-type calcium channel-dependent neuronal excitation in rats. *J. Physiol. Pharmacol.* 63 61–68. 22460462

[B21] Miyamoto R. Otsuguro K. Yamaguchi S. Ito S. (2015). Neuronal regulation of expression of hydrogen sulfide-producing enzyme cystathionine beta-synthase in rat spinal cord astrocytes. *Neurosci. Res.* 97 52–59. 10.1016/j.neures.2015.03.003 25797494

[B22] Qu K. Lee S. W. Bian J. S. Low C. M. Wong P. T. (2008). Hydrogen sulfide: neurochemistry and neurobiology. *Neurochem. Int.* 52 155–165. 10.1016/j.neuint.2007.05.016 17629356

[B23] Qu R. B. Tao J. D. Wang Y. M. Zhou Y. L. Wu G. P. Xiao Y. (2013). Neonatal colonic inflammation sensitizes voltage-gated Na^+^ channels via upregulation of cystathionine beta-synthetase expression in rat primary sensory neurons. *Am. J. Physiol. Gastrointest. Liver Physiol.* 304 G763–G772. 10.1152/ajpgi.00466.2012 23449670

[B24] Ruscheweyh R. Sandkuhler J. (2002). Lamina-specific membrane and discharge properties of rat spinal dorsal horn neurones in vitro. *J. Physiol.* 541(Pt 1) 231–244. 12015432 10.1113/jphysiol.2002.017756 PMC2290304

[B25] Sandler R. S. Drossman D. A. Nathan H. P. McKee D. C. (1984). Symptom complaints and health care seeking behavior in subjects with bowel dysfunction. *Gastroenterology* 87 314–318. 6735075

[B26] Sha L. Linden D. R. Farrugia G. Szurszewski J. H. (2013). Hydrogen sulfide selectively potentiates central preganglionic fast nicotinic synaptic input in mouse superior mesenteric ganglion. *J. Neurosci.* 33 12638–12646. 10.1523/JNEUROSCI.4429-12.2013 23904600 PMC3728682

[B27] Syhr K. M. J. Boosen M. Hohmann S. W. Longen S. Kohler Y. Pfeilschifter J. (2015). The H2S-producing enzyme CSE is dispensable for the processing of inflammatory and neuropathic pain. *Brain Res.* 1624 380–389. 10.1016/j.brainres.2015.07.058 26271715

[B28] Tan B. H. Wong P. T. Bian J. S. (2010). Hydrogen sulfide: a novel signaling molecule in the central nervous system. *Neurochem. Int.* 56 3–10. 10.1016/j.neuint.2009.08.008 19703504

[B29] Velasco-Xolalpa M. E. Barragan-Iglesias P. Roa-Coria J. E. Godinez-Chaparro B. Flores-Murrieta F. J. Torres-Lopez J. E. (2013). Role of hydrogen sulfide in the pain processing of non-diabetic and diabetic rats. *Neuroscience* 250 786–797. 10.1016/j.neuroscience.2013.06.053 23830907

[B30] Wang Y. M. Qu R. B. Hu S. F. Xiao Y. Jiang X. H. Xu G. Y. (2012). Upregulation of cystathionine beta-synthetase expression contributes to visceral hyperalgesia induced by heterotypic intermittent stress in rats. *PLOS ONE* 7:e53165 10.1371/journal.pone.0053165 PMC3532424 23285261

[B31] Winston J. Shenoy M. Medley D. Naniwadekar A. Pasricha P. J. (2007). The vanilloid receptor initiates and maintains colonic hypersensitivity induced by neonatal colon irritation in rats. *Gastroenterology* 132 615–627. 10.1053/j.gastro.2006.11.014 17258716

[B32] Wu S. X. Wang W. Li H. Wang Y. Y. Feng Y. P. Li Y. Q. (2010). The synaptic connectivity that underlies the noxious transmission and modulation within the superficial dorsal horn of the spinal cord. *Prog. Neurobiol.* 91 38–54. 10.1016/j.pneurobio.2010.01.005 20100541

[B33] Xie R. G. Gao Y. J. Park C. K. Lu N. Luo C. Wang W. T. (2017). Spinal CCL2 promotes central sensitization, long-term potentiation, and inflammatory pain via CCR2: further insights into molecular, synaptic, and cellular mechanisms. *Neurosci. Bull.* 10.1007/s12264-017-0106-5 [Epub ahead of print]. PMC5587365 28265898

[B34] Xu G. Y. Shenoy M. Winston J. H. Mittal S. Pasricha P. J. (2008). P2X receptor-mediated visceral hyperalgesia in a rat model of chronic visceral hypersensitivity. *Gut* 57 1230–1237. 10.1136/gut.2007.134221 18270243

[B35] Xu G. Y. Winston J. H. Shenoy M. Zhou S. Chen J. D. Pasricha P. J. (2009). The endogenous hydrogen sulfide producing enzyme cystathionine-beta synthase contributes to visceral hypersensitivity in a rat model of irritable bowel syndrome. *Mol. Pain* 5:44 10.1186/1744-8069-5-44 PMC2731739 19660142

[B36] Yan J. Hu S. Zou K. Xu M. Wang Q. Miao X. (2016). Inhibition of cystathionine beta-synthetase suppresses sodium channel activities of dorsal root ganglion neurons of rats with lumbar disc herniation. *Sci. Rep.* 6:38188 10.1038/srep38188 PMC5131276 27905525

[B37] Yang H. Y. Wu Z. Y. Bian J. S. (2014). Hydrogen sulfide inhibits opioid withdrawal-induced pain sensitization in rats by down-regulation of spinal calcitonin gene-related peptide expression in the spine. *Int. J. Neuropsychopharmacol.* 17 1387–1395. 10.1017/S1461145714000583 24824948

[B38] Yang K. Li Y. Kumamoto E. Furue H. Yoshimura M. (2001). Voltage-clamp recordings of postsynaptic currents in substantia gelatinosa neurons in vitro and its applications to assess synaptic transmission. *Brain Res. Brain Res. Protoc.* 7 235–240. 10.1016/S1385-299X(01)00069-1 11431124

[B39] Yang K. Li Y. Q. (2000). Postsynaptic origin of N-methyl-D-aspartate-induced slow currents in substantia gelatinosa neurons: an in vitro voltage-clamp study in adult rat. *Neurosci. Lett.* 292 21–24. 10.1016/S0304-3940(00)01426-9 10996440

[B40] Yasaka T. Kato G. Furue H. Rashid M. H. Sonohata M. Tamae A. (2007). Cell-type-specific excitatory and inhibitory circuits involving primary afferents in the substantia gelatinosa of the rat spinal dorsal horn in vitro. *J. Physiol.* 581(Pt 2) 603–618. 10.1113/jphysiol.2006.123919 17347278 PMC2075204

[B41] Yoshimura M. Jessell T. M. (1989). Membrane properties of rat substantia gelatinosa neurons in vitro. *J. Neurophysiol.* 62 109–118. 2754464 10.1152/jn.1989.62.1.109

[B42] Yoshimura M. Nishi S. (1993). Blind patch-clamp recordings from substantia gelatinosa neurons in adult rat spinal cord slices: pharmacological properties of synaptic currents. *Neuroscience* 53 519–526. 10.1016/0306-4522(93)90216-3 8098516

[B43] Zhang H. H. Hu J. Zhou Y. L. Hu S. Wang Y. M. Chen W. (2013). Promoted interaction of nuclear factor-kappaB with demethylated cystathionine-beta-synthetase gene contributes to gastric hypersensitivity in diabetic rats. *J. Neurosci.* 33 9028–9038. 10.1523/JNEUROSCI.1068-13.2013 23699514 PMC6705038

[B44] Zhou L. J. Liu X. G. (2017). Glial activation, a common mechanism underlying spinal synaptic plasticity? *Neurosci. Bull.* 33 121–123. 10.1007/s12264-016-0091-0 27995566 PMC5567553

